# PERIOD–TIMELESS Interval Timer May Require an Additional Feedback Loop

**DOI:** 10.1371/journal.pcbi.0030154

**Published:** 2007-08-03

**Authors:** Robert S Kuczenski, Kevin C Hong, Jordi García-Ojalvo, Kelvin H Lee

**Affiliations:** 1 School of Chemical and Biomolecular Engineering, Cornell University, Ithaca, New York, United States of America; 2 Departament de Física i Enginyeria Nuclear, Universitat Politècnica de Catalunya, Terrassa, Spain; University of California San Diego, United States of America

## Abstract

In this study we present a detailed, mechanism-based mathematical framework of *Drosophila* circadian rhythms. This framework facilitates a more systematic approach to understanding circadian rhythms using a comprehensive representation of the network underlying this phenomenon. The possible mechanisms underlying the cytoplasmic “interval timer” created by PERIOD–TIMELESS association are investigated, suggesting a novel positive feedback regulatory structure. Incorporation of this additional feedback into a full circadian model produced results that are consistent with previous experimental observations of wild-type protein profiles and numerous mutant phenotypes.

## Author Summary

The ability of an organism to adapt to daily changes in the environment, via a circadian clock, is an inherently interesting phenomenon recently connected to several human health issues. Decades of experiments on one of the smallest model animals, the fruit fly *Drosophila,* has illustrated significant similarities with the mammal circadian system. Within *Drosophila*, the PERIOD and TIMELESS proteins are central to controlling this rhythmicity and were recently shown to have a rapid and stable association creating an “interval” timer in the cell's cytoplasm. This interval timer creates the necessary delay between the expression and activity of these genes, and is directly opposed to the previous hypothesis of a delay created by slow association. We use several mathematical models to investigate the unknown factors controlling this timer. Using a novel positive feedback loop, we construct a circadian model consistent with the interval timer and many wild-type and mutant experimental observations. Our results suggest several novel genes and interactions to be tested experimentally.

## Introduction

**Figure 1:**
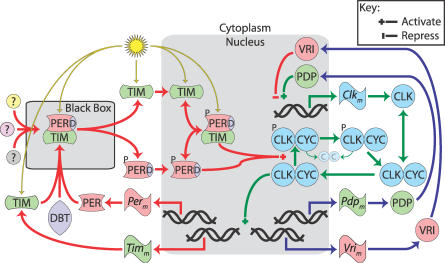
A Detailed Framework of Circadian Rhythms in *Drosophila*

Circadian rhythmicity is the product of a robust [1], free-running, temperature-compensated [2], and adaptable [3,4] biological clock found in diverse organisms ranging from bacteria to humans. The model organism *Drosophila* is commonly used to study this phenomenon due to the relative ease of experimentation and the similarities to the mammalian circadian clock (reviewed in [5,6]). The *Drosophila* circadian clock is composed of two interlocking feedback loops, shown in Figure 1. The first loop is composed of the negative feedback of *period* (*per*) and *timeless* (*tim*), shown in red, which down-regulate their own expression by inhibiting the CLOCK–CYCLE (CLK–CYC) transcription factor. DOUBLE-TIME (DBT) binds to and phosphorylates PER, which dimerizes with TIM before localizing to the nucleus via an uncharacterized mechanism. Circadian rhythms are entrained by light through an increased degradation of TIM protein, shown in yellow. In the second loop, shown in blue, the expression of *clk* is regulated by *vrille* (*vri*) and *PAR domain protein 1 isoform ɛ* (*pdp*). Both *vri* and *pdp* expression are activated by CLK–CYC. VRI represses the expression of *clk*, creating a negative feedback loop, whereas PDP creates a positive feedback loop through activating *clk* expression. Incorporating detail on interlocked feedback loops, recently shown to increase the stability and robustness of oscillations [7,8], may be important to accurately capture the network behavior.

Several mathematical models have been created to better characterize the network underlying circadian rhythmicity in *Drosophila* (e.g., [9–14]). These initial studies provided important insights into the molecular mechanisms of circadian rhythms and the ability to produce robust 24-hour oscillations. However, recent experimental observations have created a more detailed view of network interactions, including new critical aspects that are not described by previous models. It is thus necessary to establish whether a mathematical model of the current consensus network would provide robust oscillations.

The nuclear localization of PER and TIM and the subsequent repression of CLK activity have been two particularly active areas of experimental research. The necessity of TIM for PER nuclear localization has been long established [15] and was assumed to occur through the nuclear transport of PER–TIM dimers. In contrast to this mechanism, recent experimental observations now suggest that PER and TIM localize to the nucleus in a primarily independent mechanism [16–21]. Additionally, while TIM is required for PER nuclear localization via a cytoplasmic “interval timer,” the mechanism controlling this timer is independent of both TIM and PER concentration [21]. Thus, the way in which TIM affects PER nuclear localization is an open question. Once in the nucleus, PER (and to a much lesser extent TIM) repress CLK activity, recently observed to occur via PER-mediated phosphorylation of CLK by DBT [22,23]. These studies also provided evidence that total levels of CLK remain nearly constant [22,23], in contrast to previously observed CLK oscillations [24–26]. It remains unclear whether constant total CLK concentration can coincide with stable oscillations in this new network.

To address these questions, we first study the possible mechanisms underlying the PER/TIM cytoplasmic interaction in *Drosophila* S2 cell culture using mathematical models of this isolated (arrhythmic) network. Using the most likely candidate mechanism, one based on positive feedback, we created a detailed mathematical model of the wild-type *Drosophila* circadian network. This model incorporates post-translational modifications to the PER and CLK proteins in addition to including both interlocked feedback loops, without the use of explicit time delays. The results of this model are consistent with wild-type and mutant experimental observations, provide insight into recent network revisions, and suggest possible experimental directions to explore.

## Results

### Isolation of *per*/*tim* Feedback Loop

**Figure 2:**
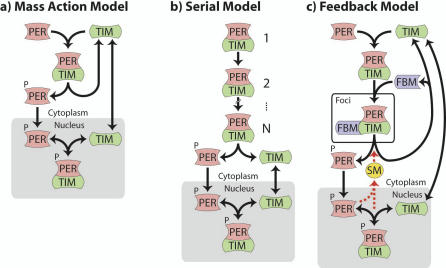
Models of the Isolated *per*/*tim* Loop (A) The simple mass action kinetics model. (B) The serial model is based on a series of intermediate (possibly phosphorylated) PER–TIM states. (C) The feedback model proposes a new role for PER providing positive feedback on the dissociation of cytoplasmic PER–TIM complexes.

To investigate the six-hour delay created by the cytoplasmic interval timer observed in S2 cell culture by Meyer et al. [21], the dynamics of the *per*/*tim* loop were isolated and studied independently of the remaining circadian gene network to mimic the environment within *Drosophila* S2 cells. The interactions constituting the three mathematical models studied are shown in Figure 2. All models of the isolated *per*/*tim* loop include PER–TIM dimers in the cytoplasm that dissociate immediately prior to nuclear localization and re-association, but differ in the mechanism controlling the timing of this dissociation.

**Figure 3:**
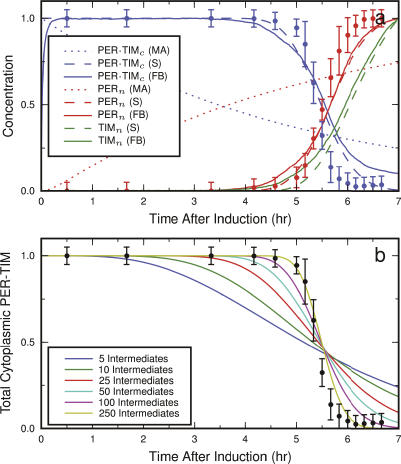
Results of the Isolated *per*/*tim* Loop Models versus Experimental Data Adapted from Meyer et al. [21] (Points with Error Bars) (A) Five hours after induction, the isolated *per*/*tim* loop from the simple mass action (MA) model results show PER is nuclear but without the precipitous change in PER–TIM stability and PER localization. Both the serial (S) (100 intermediates) and the feedback (FB) model results are consistent with experimental observations, showing a precipitous dissociation or PER–TIM followed by the nuclear accumulation of PER and TIM. (B) The serial model requires hundreds of intermediate states to be consistent with experimental observations. Model results and experimental data were scaled to a maximum of 1.0. Subscripts denote cytoplamic (*c*) or nuclear (*n*) localization.

The mass action model is the simplest isolated model and is based on the commonly accepted *per*/*tim* interactions shown in Figure 2A. In this model, PER–TIM dimers simply dissociate prior to independent nuclear transport. The dynamics of this model, shown as dotted lines in Figure 3A, were able to produce nuclear localization of PER six hours after inducing expression, but did not accurately capture the switch-like dissociation of PER–TIM observed experimentally [21].

Next, a model based on the sequential modification of PER–TIM dimers, termed the serial model, was created as shown in Figure 2B. The serial mechanism may represent the sequential phosphorylation of PER and/or TIM. To simplify the mathematics of this model (see Materials and Methods), PER–TIM dimers were assumed to be initially associated before the proceeding series of modifications after which nuclear localization occurs. Interestingly, this model required hundreds of intermediates to produce a stable five-hour association followed by a precipitous dissociation, as shown in Figure 3B.

**Figure 4:**
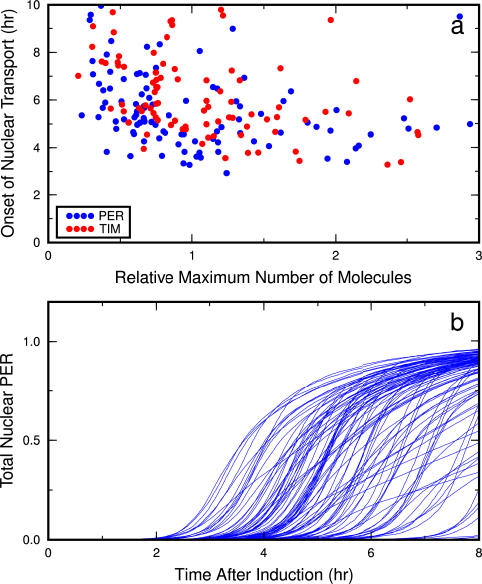
Nuclear Onset in the Isolated Positive Feedback Model (A) The onset of PER nuclear localization versus the maximum concentration of PER (blue) and TIM (red). The initial concentrations of PER and TIM were varied according to a log normal distribution (see Materials and Methods) in the stochastic implementation of the positive feedback isolated *per*/*tim* loop model. The onset of nuclear localization is largely independent of PER and TIM concentration, consistent with experimental observations [21]. (B) The ensemble of total nuclear PER trajectories used to create the blue dots in (A). Trajectories were scaled to a maximum of 1.0.

Finally, a model based on positive feedback (previously suggested to increase clock accuracy via the PDP loop of the full circadian network [27,28]) was created as shown in Figure 2C. Consistent with experimental observations [21], this model explicitly represents the cytoplasmic association of PER–TIM dimers and the subsequent localization of these dimers into discrete foci. Within the foci, a background level of activity creates a low amount of dissociation and PER nuclear localization. A nuclear-generated signaling molecule (SM) is then created in response to PER and is used to complete the positive feedback on the dissociation of PER–TIM in foci. This network is conceptually consistent with the observation that blocking nuclear export (and thus preventing the SM in this model from exiting the nucleus and exerting the positive feedback) delays nuclear localization [21]. The timing of PER nuclear localization in this model, shown as solid lines in Figure 3A, is consistent with experimental observations [21]. In addition to the feedback SM, this model incorporates another unknown component: the focus-binding mediator (FBM) molecule. The presence of this molecule at limiting concentrations creates a nuclear localization timer that is largely independent of the maximum PER and TIM concentration, as shown in Figure 4A.

### Wild-Type Observations

**Figure 5:**
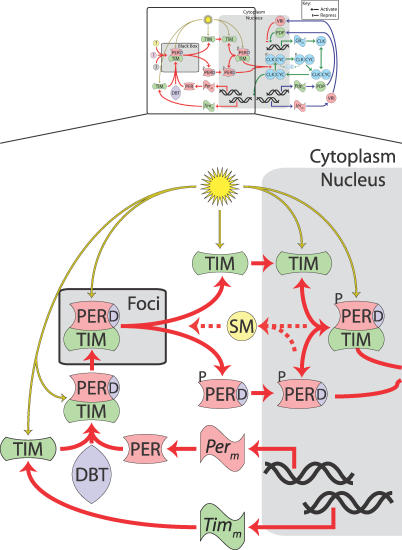
Detailed Model Black Box The *per*/*tim* loop of the detailed mathematical model of *Drosophila* circadian rhythmicity, a possible mechanism underlying the black box in Figure 1. Only one type of monomeric TIM and no monomeric DBT species were explicitly represented in the model. See Materials and Methods for a list of model equations.

**Figure 6:**
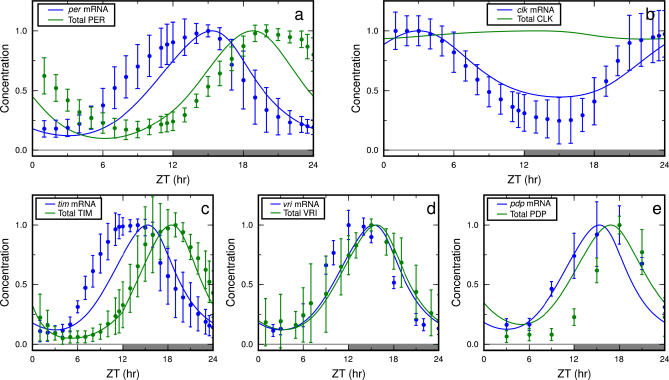
Comparison of the Results with the Experimental Data The expression profiles of *per* (A), *clk* (B), *tim* (C), *vri* (D), and *pdp* (E) mRNA and total protein from the detailed model results (lines) were compared with experimental observations (points with error bars generated from the average and standard deviation of published experimental data, see Table S4). Model results and experimental data were normalized to 1.0. Bars along the horizontal axis indicate light entrainment regime: light-on (empty) or light-off (filled).

A model of the full circadian network was created based on a simplification of the positive feedback isolated *per*/*tim* loop model, the interactions of which are shown in Figures 1 and 5. The expression of *per*, *tim*, *clk*, *vri*, and *pdp* mRNA and total protein are in excellent agreement with experimental observations, as shown in Figure 6 (see references therein). The model results show a period of 24.0 hours under a light–dark cycle (Figure 6) and 23.8 hours in constant darkness, also consistent with experimental observations.

### Gene Dosage Affects Period Length

**Figure 7:**
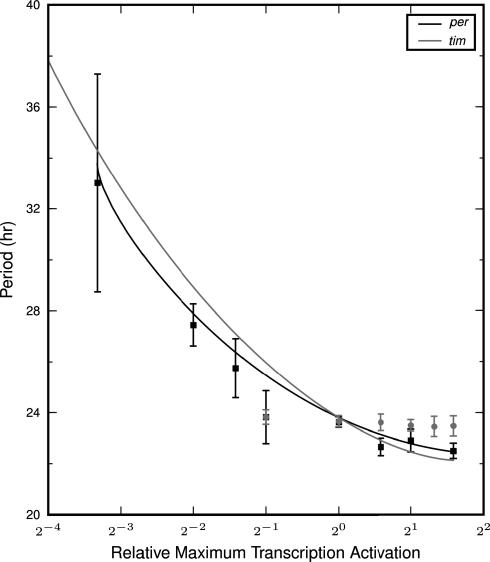
The Effect of Gene Dosage on the Period of Oscillation in Constant Darkness A continuation analysis of *per* dosage-dependent behavior of period under constant darkness (black line) shows an inverse relation between the maximum transcription activation of *per* and the period length, which is consistent with the experimental results (represented by black squares and error bars) [29,30]. A continuation analysis of *tim* dosage-dependent behavior of period under constant darkness (gray line) shows a similar trend to *per* dosage, which is inconsistent with experimental observations (represented by gray circles and error bars) [17,31].

Our results show a *per* dosage dependence of the period length that is consistent with experimental observations [29,30]. A continuation analysis of the maximum transcriptional activation of *per* in the model demonstrates an inverse relation between *per* dosage and the period of circadian oscillation (black lines and points in Figure 7). In contrast, a continuation analysis of the maximum transcriptional activation of *tim* (gray lines and points in Figure 7) revealed a profile which is similar to *per* dosage and thus not very consistent with experimental observations [17,31].

### Mutant Phenotypes

The results from the model are consistent with numerous homozygous mutant phenotypes, as shown in Table 1 (see references therein). These results show that arrhythmic null mutants in the *per*/*tim* feedback loop (i.e., *per* ^01^ and *tim* ^01^) are unable to repress the activity of CLK–CYC resulting in constitutively high expression of unaltered *per* [32,33], *tim* [24,26,33–35], *vri* [36,37], and *pdp* [36]. The decreased PER degradation in *dbt^P^* and *dbt^ar^* mutants resulted in the stable repression of CLK–CYC activity and the constitutively low expression of *per*, *tim*, *vri*, and *pdp* mRNA and protein [34,38]. Similarly, when the level of active CLK–CYC is reduced by a knockout of CLK or CYC (*clk^Jrk^* and *cyc* ^0^) or eliminating the activator of *clk* expression (*pdp^P^* ^205^), the resulting levels of *per, tim, vri,* and *pdp* mRNA and protein are constitutively low [36,37,39–41]. Understanding the effects of these mutants provides key insights into the roles of specific genes in the network, and reproducing their behavior provides support for the model representation.

**Table 1:**
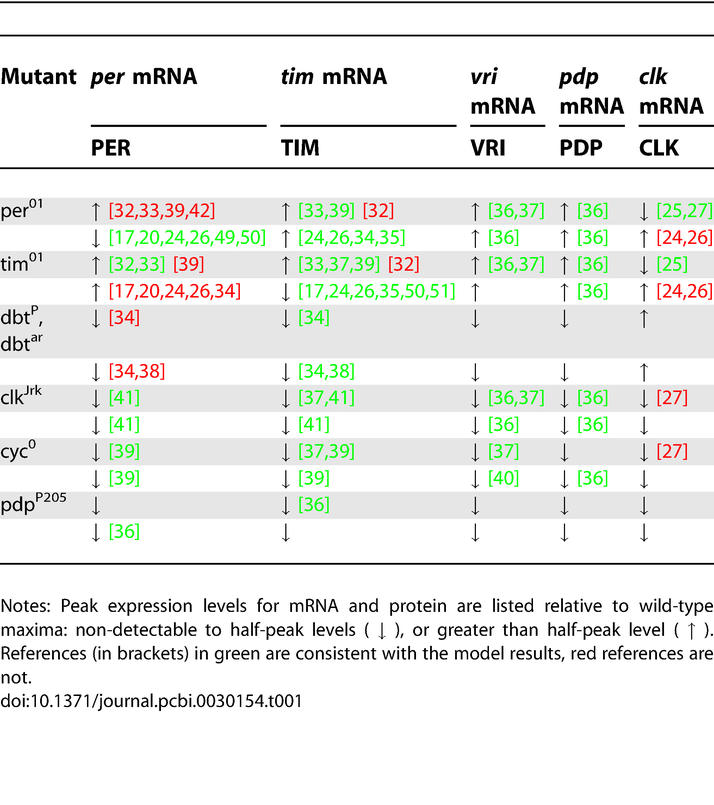
Comparison of Experimental Homozygous Arrhythmic Mutant Phenotypes

The model accurately captures a majority of the published experimental observations. However, a number of mutant flies display behavior that is not completely consistent with the model results. For example, the low levels of *tim* mRNA in *per* ^01^, *per* mRNA in *tim* ^01^, and *tim* mRNA in *tim* ^01^ from some publications [32,39] conflict with model results; however, experimental results from other publications on these same species do agree with our model results [32,33,37,39]. The low levels of *per* mRNA in *per* ^01^ [32,33,39,42], low levels of PER in *tim* ^01^ [17,20,24,26,34], and high levels of *per* mRNA and protein in *dbt^P^*/*dbt^ar^* [34,38] observed experimentally conflict with the model results and experimental observations of other E-box mRNA and protein levels. The mathematical model lacks ability to produce nuclear CLK–CYC in *clk^Jrk^* and *cyc* ^0^ mutants, breaking the activation of E-box genes and producing no *clk* mRNA in contrast to experimental observations [27]. Also, the non–PER-mediated CLK phosphorylation in the model results are not able to produce low CLK levels [24,26] without nuclear PER in the *per* ^01^ and *tim* ^01^ mutants.

## Discussion

### Interval Timer Control

The isolated mass action model results (dotted lines in Figure 3A) are not consistent with the experimental observation of stably associated PER–TIM dimers and precipitous nuclear localization [21]. The serial model results (Figure 3B) show that hundreds of intermediates may be required to produce this behavior. This number of intermediates is larger than the potential phosphorylation sites on PER and TIM predicted by ScanProsite (22 Casein Kinase II sites on PER, 32 sites on TIM) [43]. The progressive phosphorylation of PER and/or TIM may be observed as a change in electrophoretic mobility prior to nuclear localization in S2 cells. The positive feedback mechanism (solid lines in Figure 3A) is able to produce the correct delay and rapid dissociation, making it an attractive alternative to the serial model.

The FBM in the positive feedback model, for which no direct experimental evidence currently exists, is responsible for controlling the onset of nuclear localization independent of PER and TIM concentrations. Without this molecule, the onset would be well correlated to experiment-to-experiment variability in the limiting concentration of PER and/or TIM (unpublished data). The *shaggy* (*sgg*) kinase is a potential candidate because it has been previously shown to phosphorylate TIM, affecting the nuclear localization of PER [20,44], and also bind to cytoskeletal elements [45], a possible location of the cytoplasmic foci. A *sgg* knockout in S2 cells could be used to observe the possible disruption of PER/TIM accumulation in cytoplasmic foci, which would be consistent with this hypothesized role for *sgg*.

No obvious candidate for the SM exists in the literature. Because small molecules have been shown to cause significant structural changes in PAS domains [46], one possibility may be a small molecule binding to and elucidating a temporary conformational change in PER, allowing it to dissociate from TIM and localize to the nucleus.

The feedback model is not consistent with all the data presented by Meyer et al. [21]. The rates of nuclear localization of PER and TIM are not completely independent (unpublished data), and the conflict between rapid nuclear transport and well-controlled timing of nuclear localization results in a timing error that is double the observed seventy minutes [21]. These differences may be the result of additional regulatory structures not already identified.

### CLK Oscillation

The full network results demonstrate that while total CLK levels do not change significantly during the course of a day, the oscillating phosphorylation of CLK can lead to significant and stable oscillations in mRNA (see Figure 6). These near-constant total CLK levels are generated by synchronized translation and degradation (see Figure S1). This result differs from previous mathematical models [11,12,14] which show a significant oscillation in CLK level (consistent with prior experimental observations [24–26]), and suggest that the oscillation of CLK activity, not concentration, is necessary for circadian rhythmicity.

### Conclusion

We find that independent transfer of PER and TIM by simple mass action kinetics is inconsistent with experimental observations, but that an additional feedback loop (or alternatively a large number of intermediate phosphorylated states) is able to produce the switch-like dissociation of cytoplasmic PER–TIM underlying the interval timer [21]. This positive feedback was introduced into a mechanistic mathematical framework for *Drosophila* circadian rhythms which demonstrates excellent agreement with experimentally observed expression profiles of circadian genes and many circadian mutants. The framework is consistent with observations of the relationship between *per* dosage and circadian periodicity. Post-translational regulation is addressed, including the effect of phosphorylation on the transcriptional activation activity of CLK. Our results also show that the nuclear translocation of the PER and TIM can occur independently while producing stable oscillations when positive feedback is employed.

## Materials and Methods

With the exception of transcription activation kinetics, discussed below, all reaction kinetics are mass action. These kinetics were chosen for simplicity and because no direct experimental evidence is available suggesting other (e.g*.,* Michaelis-Menten or Hill) kinetic forms. Unless otherwise noted, molecules use subscripts to denote mRNA (m), free cytoplasmic protein (c), focus-bound protein (f), and nuclear protein (n). Additionally, dimers are represented as [*X* · *Y*] and phosphorylated isoforms as [*X* · *P*]. All models are available in SBML format in Protocols S1–S4.

### Isolated *per*/*tim* loop models.

The simple mass action model of the isolated *per*/*tim* loop is represented by Equations 1–7 below.

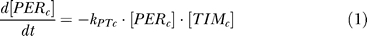


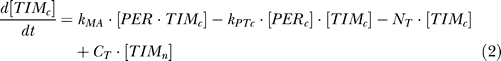


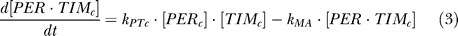


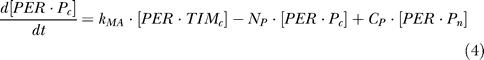


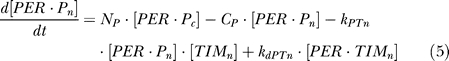


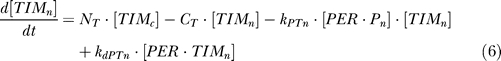


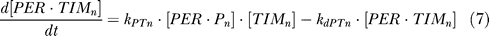



The serial model is represented by Equations 8–13 below, where *N* is the number of intermediates in the reaction series.

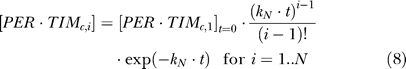


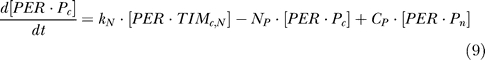


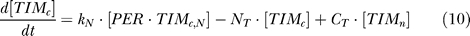


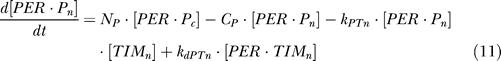


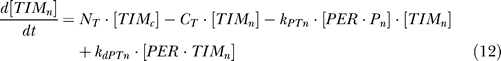


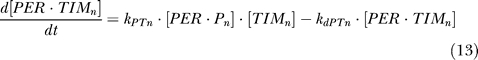



To simplify the solution of the serial model, initial concentrations of monomeric PER and TIM in the cytoplasm were eliminated by assuming that their dimerization occurred quickly. This assumption allowed the analytic solution of the last PER–TIM dimer in the series of N reactions, and greatly reduced the number of equations for large N.

The positive feedback model is represented by Equations 14–23 below. To represent the concentration effect of foci localization, a second-order term is used for slow dissociation of PER–TIM from the foci (see Equations 15, 17, and 18). Additionally, SM is assumed to catalyze the release of PER–TIM from the foci, and thus is not depleted by this reaction.

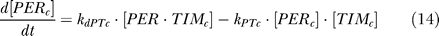


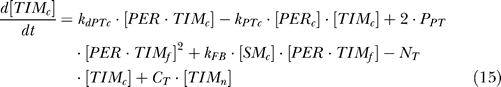


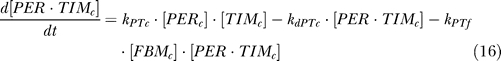


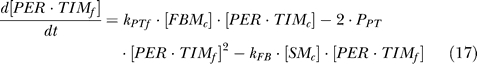


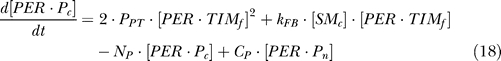


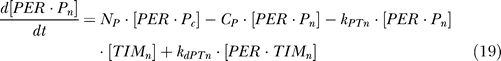


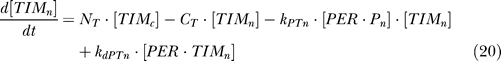


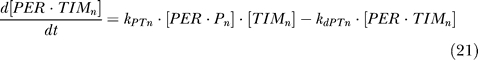


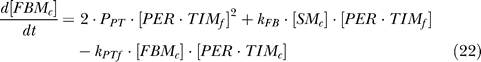


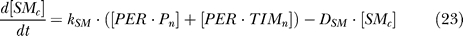



The initial concentrations of cytoplasmic PER and TIM in the mass action and positive feedback models and the first PER–TIM dimer in the serial model were set to the maximum concentration of PER and TIM (10,000 molecules or approximately 10^4^ nM). The initial concentration of FBM in the positive feedback model was set to 5,000 molecules. All other initial concentrations in the isolated models were set to zero. Additionally, the initial conditions of PER and TIM in the positive feedback model were varied in magnitude based on a log normal distribution fit to the data presented in Figure 1C of [21]. See Table S1 for a full list of reaction rate constants for the isolated models.

### Detailed mathematical model.

A detailed mathematical framework of *Drosophila* circadian rhythms using the differential equations below was created based on the interactions shown in Figures 1 and 5.

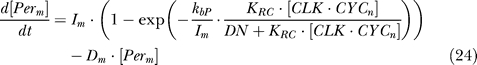


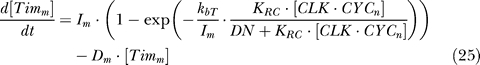


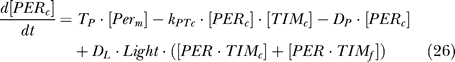


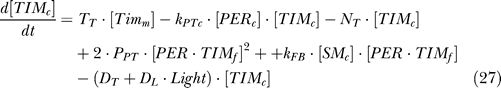


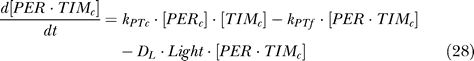


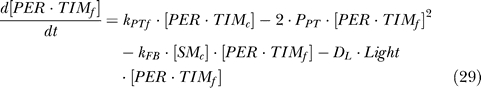


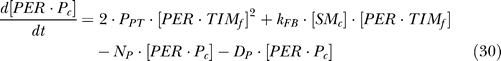


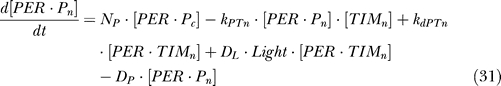


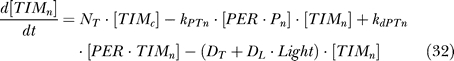


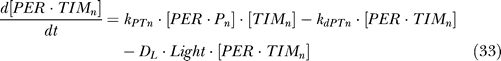


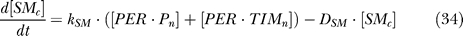


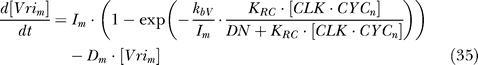


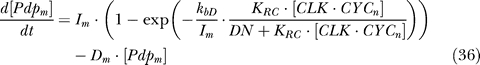


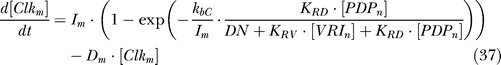


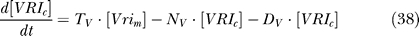


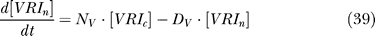


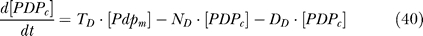


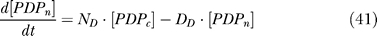


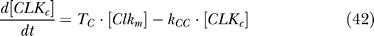


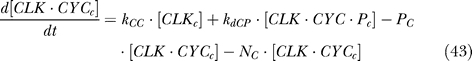


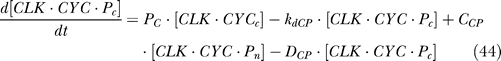


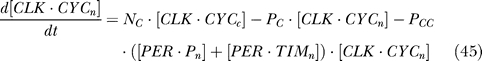


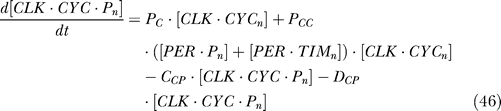


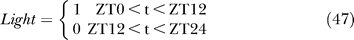



This description does not use time delays and explicitly represents the post-translational modifications of PER and CLK. Illumination in light–dark cycles is modeled via *Light,* defined as a square wave in Equation 47. Light acts upon the degradation of cytoplasmic and nuclear TIM. The transcriptional activation kinetics are borrowed from [47], and described in Text S1. FBM is not explicitly represented because the inclusion of this molecule at limiting concentrations did not significantly alter the presented results (unpublished data). As shown in Figure 1 (and based on the observations of [22,23]), the presence of nuclear PER and PER–TIM dimers causes the phosphorylation of CLK. Once phosphorylated, CLK cannot bind to DNA and is either degraded or exported into the cytoplasm where it can be degraded or dephosphorylated. Chemical reaction rate constants are the only adjustable parameters for which a set of biologically meaningful values was found (see Parameter Estimation below). See Table S2 for a full list of reaction rate constants for the full circadian model.

### Model solutions.

With the exception of the positive feedback model of the isolated *per*/*tim* loop, the mathematical models presented in this paper were solved using the LSODAR integrator as part of the SloppyCell package [48]. Periodic orbits were found through sequential integration cycles until a stable limit cycle was approached. For the continuation analysis of model parameters, AUTO 2000 was used.

Since a small number of molecules may initiate positive feedback, the isolated *per*/*tim* loop positive feedback model was solved stochastically using Gillespie's algorithm. The model results are an ensemble of trajectories for a given parameter set (a randomly selected subset which is shown in Figure 4B), with the trajectory closest to the experimentally observed mean nuclear onset time used in Figure 3A. The standard deviation of nuclear onset time was determined from this ensemble of trajectories.

### Mutant phenotype characterization.

Several *Drosophila* mutant phenotypes were represented by the detailed mathematical model. The parameter changes used to represent the mutants described in the paper are shown in Table S3. A typical result is shown for the arrhythmic *dbt^p^*/*dbt^ar^* mutants in Figure S2. The transient trajectory from a point on the wild-type constant darkness limit cycle illustrates the approach to a stable fixed point solution. Similarly, all arrhythmic mutants presented in Table 1 were found to approach stable fixed points (unpublished data).

### Experimental data.

The points and error bars presented in Figure 3 were the result of averaging the five trajectories for cytoplasmic PER–TIM dimers and nuclear PER shown in Figure 1B of Meyer et al. [21]. These trajectories were normalized to a minimum of zero and maximum of one prior to aligning the paired PER–TIM and nuclear PER trajectories by minimizing the root mean-squared distance. The mean onset time of the average of the aligned trajectories was then set to 340 min.

The points and error bars in Figure 6 were adapted from the publications listed in Table S4. With the exception of *pdp* mRNA and protein, multiple references were available. These datasets were interpolated and averaged to produce the means and standard deviations presented in Figure 6. *pdp* mRNA and protein means and error bars were taken directly from [36].

The points and error bars in Figure 7 are the average and standard deviation of experimental observations of the period of oscillation in response to changes in *per* dosage [29,30] and *tim* dosage [17,31].

### Parameter estimation.

A Monte Carlo random walk, guided by importance sampling, adjusted model parameters to optimize a chi-squared value quantifying the consistency of the model results with available experimental observations (discussed in the previous section and presented in Figures 3, 6, and 7). Model parameters were manually adjusted to biologically meaningful values where necessary.

## Supporting Information

Figure S1 The Translation or Degradation of *clk* Protein Balance To Produce a Nearly Constant Level of Total CLK The reaction rate shown is normalized by the maximum level of total CLK. The degradation term includes both cytoplasmic and nuclear degradation (as defined in Equations 44 and 46).
(7.3 MB TIF)

Figure S2 The Evolution of the *dbt^P^*/*dbt^ar^* Mutants away from a Point on the Wild-Type Constant Darkness Limit Cycle (319 KB TIF)

Protocol S1 SBML Representation of the Isolated Mass Action Model (7 KB TXT)

Protocol S2 SBML Representation of the Isolated Serial Model for N = 100 (111 KB TXT)

Protocol S3 SBML Representation of the Isolated Positive Feedback Model (10 KB TXT)

Protocol S4 SBML Representation of the Detailed Circadian Rhythms Model (51 KB TXT)

Table S1 Isolated Model Parameters (33 KB DOC)

Table S2 Full Circadian Model Parameters (50 KB DOC)

Table S3 Parameter Changes to Describe Mutant Phenotypes (28 KB DOC)

Table S4 Experimental mRNA and Protein Measurement Sources (31 KB DOC)

Text S1 Derivation of Transcription Activation Terms (32 KB DOC)

## Abbreviations

*clk*: *clock*
*cyc*: *cycle*
*dbt*: *double-time*
FBM: focus-binding mediator
*pdp*: *PAR domain protein 1 isoform *ɛ
*per*: *period*
SM: signaling molecule
*tim*: *timeless*
*vri*: *vrille*
ZT: zeitgeber time
