# Supplementary material for: PERIOD–TIMELESS Interval Timer May Require an Additional Feedback Loop
Source: PLoS Comput Biol. 2007 Aug 3;3(8):e154. doi: 10.1371/journal.pcbi.0030154 (PMC1937016; doi:10.1371/journal.pcbi.0030154)
Supplement: Supplementary file 1 [file pcbi.0030154.sd005.doc]

### Supporting Text 1. Derivation of Transcription Activation Terms

The activation of *per*, *tim*, *vri*, and *pdp* transcription are derived as described in the Supporting Text of Bolouri and Davidson [1]. The activation of *clk* transcription by *pdp* with repression by *vri* is derived below. Similar to Bolouri and Davidson, we assume: 1) equilibrium in factor binding to both specific and non-specific sites, 2) essentially all factor is bound to either specific or non-specific sites (*i.e.* negligible free factor), and 3) total factor concentration is much greater than specific site-bound factor (or similarly that non-specific site-bound factor greatly outnumbers specific site-bound factor). Finally, we assume VRI competitively inhibits PDP binding.

$$Y=\frac{\left[PDP_{n}\cdot D_{S}\right]}{\left[D_{S}\right]+\left[VRI_{n}\cdot D_{S}\right]+\left[PDP_{n}\cdot D_{S}\right]}$$

$K_{equilibrium\_S,VRI}=\frac{\left[VRI_{n}\cdot D_{S}\right]}{\left[D_{S}\right]\cdot\left[VRI_{n}\right]}$, $K_{equilibrium\_N,VRI}=\frac{\left[VRI_{n}\cdot D_{N}\right]}{\left[D_{N}\right]\cdot\left[VRI_{n}\right]}$

$$K_{RV}=\frac{K_{equilibrium\_S,VRI}}{K_{equilibrium\_N,VRI}}=\frac{\left[VRI_{n}\cdot D_{S}\right]}{\left[D_{S}\right]}\cdot\frac{\left[D_{N}\right]}{\left[VRI_{n}\cdot D_{N}\right]}$$

Similarly for PDP binding:

$$K_{RD}=\frac{K_{equilibrium\_S,PDP}}{K_{equilibrium\_N,PDP}}=\frac{\left[PDP_{n}\cdot D_{S}\right]}{\left[D_{S}\right]}\cdot\frac{\left[D_{N}\right]}{\left[PDP_{n}\cdot D_{N}\right]}$$

$$Y=\frac{K_{RD}\cdot\left[PDP_{n}\cdot D_{N}\right]}{\left[D_{N}\right]+K_{RV}\cdot\left[VRI_{n}\cdot D_{N}\right]+K_{RD}\cdot\left[PDP_{n}\cdot D_{N}\right]}$$

Applying assumptions 2 and 3 yields:

$$\left[VRI_{n}\right]=\left[VRI_{n,free}\right]+\left[VRI_{n}\cdot D_{S}\right]+\left[VRI_{n}\cdot D_{N}\right]=\left[VRI_{n}\cdot D_{N}\right]$$

$$\left[PDP_{n}\right]=\left[PDP_{n,free}\right]+\left[PDP_{n}\cdot D_{S}\right]+\left[PDP_{n}\cdot D_{N}\right]=\left[PDP_{n}\cdot D_{N}\right]$$

$$Y=\frac{K_{RD}\cdot\left[PDP_{n}\right]}{\left[D_{N}\right]+K_{RV}\cdot\left[VRI_{n}\right]+K_{RD}\cdot\left[PDP_{n}\right]}$$

The final kinetic term is derived as described in the Supplemental Text of Bolouri and Davidson [1].

### References

1. Bolouri H, Davidson EH (2003) Transcriptional regulatory cascades in development: initial rates, not steady state, determine network kinetics. Proc Natl Acad Sci U S A 100:9371–9376.
