# Supplementary material for: PERIOD–TIMELESS Interval Timer May Require an Additional Feedback Loop
Source: PLoS Comput Biol. 2007 Aug 3;3(8):e154. doi: 10.1371/journal.pcbi.0030154 (PMC1937016; doi:10.1371/journal.pcbi.0030154)
Supplement: Supplementary file 2 [file pcbi.0030154.st001.doc]

***begin of environment: TABLE ***

| **Name** | **Value** | **Description** |
| --- | --- | --- |
|  |  |  |
| *kMA* | 0.2 hr^-1^ | PER-TIM phosphorylation |
| *k*_5_ | 0.9091 hr^-1^ | Serial Reaction Coefficient (N=5) |
| *k*_10_ | 1.818 hr^-1^ | Serial Reaction Coefficient (N=10) |
| *k*_25_ | 4.545 hr^-1^ | Serial Reaction Coefficient (N=25) |
| *k*_50_ | 9.091 hr^-1^ | Serial Reaction Coefficient (N=50) |
| *k*_100_ | 18.18 hr^-1^ | Serial Reaction Coefficient (N=100) |
| *k*_250_ | 45.45 hr^-1^ | Serial Reaction Coefficient (N=250) |
| *kPTc* | 0.01 (nM*hr)^-1^ | Cytoplasmic PER-TIM association |
| *kdPTc* | 0.1 hr^-1^ | Cytoplasmic PER-TIM dissociation |
| *kPTf* | 0.0001 (nM*hr)^-1^ | Foci PER-TIM accumulation |
| *PPT* | 1.6 x 10^-7^ hr^-1^ | Foci PER-TIM auto-phosphorylation |
| *kFB* | 0.02 (nM*hr)^-1^ | Foci PER-TIM phosphorylation via feedback |
| *kSM* | 0.25 hr^-1^ | SM*c* activation |
| *DSM* | 1 hr^-1^ | SM*c* degradation |
| *NP* | 5 hr^-1^ | Phosphorylated PER nuclear transport |
| *CP* | 0.01 hr^-1^ | PER cytoplasmic transport |
| *NT* | 1 hr^-1^ | TIM nuclear transport |
| *CT* | 500 hr^-1^ | TIM cytoplasmic transport |
| *kPTn* | 1 (nM*hr)^-1^ | Nuclear PER-TIM association |
| *kdPTn* | 0.01 hr^-1^ | Nuclear PER-TIM dissociation |

***end of environment: TABLE ***
