# Supplementary material for: PERIOD–TIMELESS Interval Timer May Require an Additional Feedback Loop
Source: PLoS Comput Biol. 2007 Aug 3;3(8):e154. doi: 10.1371/journal.pcbi.0030154 (PMC1937016; doi:10.1371/journal.pcbi.0030154)
Supplement: Supplementary file 3 [file pcbi.0030154.st002.doc]

***begin of environment: TABLE ***

| **Name** | **Value** | **Description** |
| --- | --- | --- |
|  |  |  |
| *Im* | 6.027 nM/hr | Maximum transcription rate |
| *DN* | 1.363 x 10^6^ nM | Non-specific DNA binding sites |
| *KRC* | 1.372 x 10^4^ | CLK-CYC-DNA relative equilibrium constant |
| *KRV* | 1.65 x 10^9^ | VRI-DNA relative equilibrium constant |
| *KRD* | 4.007 x 10^6^ | PDP-DNA relative equilibrium constant |
| *kbP* | 54.59 nM/hr | CLK-CYC activation of *per* |
| *kbT* | 38.05 nM/hr | CLK-CYC activation of *tim* |
| *kbV* | 64.87 nM/hr | CLK-CYC activation of *vri* |
| *kbD* | 423.4 nM/hr | CLK-CYC activation of *pdp* |
| *kbC* | 29.66 nM/hr | PDP activation of *clk* |
| *Dm* | 0.2731 hr^-1^ | mRNA degradation |
| *TP* | 0.5686 hr^-1^ | PER translation rate |
| *TT* | 1.041 hr^-1^ | TIM translation rate |
| *TV* | 4.602 hr^-1^ | VRI translation rate |
| *TD* | 0.1649 hr^-1^ | PDP translation rate |
| *TC* | 0.06578 hr^-1^ | CLK translation rate |
| *kPTc* | 33.15 (nM*hr)^-1^ | Cytoplasmic PER-TIM association |
| *kPTf* | 4.092 hr^-1^ | Foci PER-TIM accumulation |
| *PPT* | 0.0009879 (nM*hr)^-1^ | Foci PER-TIM auto-phosphorylation |
| *kPTn* | 6.668 (nM*hr)^-1^ | Nuclear PER-TIM association |
| *kdPTn* | 1.483 hr^-1^ | Nuclear PER-TIM dissociation |
| *kSM* | 1.484 hr^-1^ | SM*c* activation |
| *kFB* | 1.473 (nM*hr)^-1^ | Foci PER-TIM phosphorylation via feedback |
| *kCC* | 2.497 hr^-1^ | CLK association to CYC |
| *kdCP* | 0.4666 hr^-1^ | CLK dephosphorylation |
| *NP* | 26.08 hr^-1^ | Phosphorylated PER nuclear transport |
| *NT* | 8.418 hr^-1^ | TIM nuclear transport |
| *NV* | 3.829 hr^-1^ | VRI nuclear transport |
| *ND* | 0.4847 hr^-1^ | PDP nuclear transport |
| *NC* | 0.1257 hr^-1^ | CLK nuclear transport |
| *CCP* | 3.058 hr^-1^ | Phosphorylated CLK cytoplasmic transport |
| *PCC* | 23.97 (nM*hr)^-1^ | PER-mediated CLK phosphorylation |
| *PC* | 0.01005 hr^-1^ | non-PER-mediated CLK phosphorylation |
| *DP* | 0.5892 hr^-1^ | PER degradation |
| *DT* | 1.252 hr^-1^ | TIM degradation |
| *DV* | 2.481 hr^-1^ | VRI degradation |
| *DD* | 0.4984 hr^-1^ | PDP degradation |
| *DCP* | 0.0975 hr^-1^ | Phorphorylated CLK degradation |
| *DSM* | 0.7384 hr^-1^ | SM*c* degradation |
| *DL* | 0.2276 hr^-1^ | Light-activated degradation |

***end of environment: TABLE ***
