# Supplementary material for: PERIOD–TIMELESS Interval Timer May Require an Additional Feedback Loop
Source: PLoS Comput Biol. 2007 Aug 3;3(8):e154. doi: 10.1371/journal.pcbi.0030154 (PMC1937016; doi:10.1371/journal.pcbi.0030154)
Supplement: Supplementary file 4 [file pcbi.0030154.st003.doc]

| **Mutant** | **Parameter** | **Reference** |
| --- | --- | --- |
| *per^01^* | *T_P_* = 0 hr^-1^ | [1] |
| *tim^01^* | *T_T_* = 0 hr^-1^ | [2] |
| *dbt^P^, dbt^ar^* | *D_P_* = 0.1736 hr^-1^ | [3, 4] |
| *clk^Jrk^* | *T_C_* = 0 hr^-1^ | [5] |
| *cyc^0^* | *k_CC_* = 0 hr^-1^ | [6] |
| *pdp^P205^* | *T_D_* = 0 hr^-1^ | [7] |

**References**

1.	Baylies MK, Bargiello TA, Jackson FR, Young MW (1987) Changes in abundance or structure of the *per* gene product can alter periodicity of the *Drosophila* clock. Nature 326:390–392.

2.	Myers MP, Wager-Smith K, Rothenfluh-Hilfiker A, Young MW (1996) Light-induced degradation of TIMELESS and entrainment of the *Drosophila* circadian clock. Science 271:1736–1740.

3.	Price JL, Blau J, Rothenfluh A, Abodeely M, Kloss B, et al. (1998) *double-time* is a novel *Drosophila* clock gene that regulates PERIOD protein accumulation. Cell 94:83–95.

4.	Rothenfluh A, Abodeely M, Young MW (2000) Short-period mutations of *per* affect a *double-time*-dependent step in the *Drosophila* circadian clock. Curr Biol 10:1399–1402.

5.	Allada R, White NE, So WV, Hall JC, Rosbash M (1998) A mutant *Drosophila* homolog of mammalian *Clock* disrupts circadian rhythms and transcription of *period* and *timeless*. Cell 93:791–804.

6.	Rutila JE, Suri V, Le M, So WV, Rosbash M, et al. (1998) CYCLE is a second bHLH-PAS clock protein essential for circadian rhythmicity and transcription of *Drosophila* *period* and *timeless*. Cell 93:805–814.

7.	Cyran SA, Buchsbaum AM, Reddy KL, Lin MC, Glossop NR, et al. (2003) *vrille*, *Pdp1*, and *dClock* form a second feedback loop in the *Drosophila* circadian clock. Cell 112:329–341.
