# Supplementary material for: PERIOD–TIMELESS Interval Timer May Require an Additional Feedback Loop
Source: PLoS Comput Biol. 2007 Aug 3;3(8):e154. doi: 10.1371/journal.pcbi.0030154 (PMC1937016; doi:10.1371/journal.pcbi.0030154)
Supplement: Supplementary file 5 [file pcbi.0030154.st004.doc]

| **Molecule** | **Reference(s)** |
| --- | --- |
| *per* mRNA | [1, 2, 3, 4, 5, 6] |
| PER | [2, 5, 7, 8] |
| *tim* mRNA | [3, 4, 6, 9, 10] |
| TIM | [5, 7, 8] |
| *clk* mRNA | [4, 6, 11, 12] |
| *vri* mRNA | [10, 11] |
| VRI | [11, 12] |
| *pdp* mRNA | [11] |
| PDP | [11] |

**References**

1.	Hardin PE, Hall JC, Rosbash M (1992) Circadian oscillations in *period* gene mRNA levels are transcriptionally regulated. Proc Natl Acad Sci U S A 89:11711–11715.

2.	Zeng H, Hardin PE, Rosbash M (1994) Constitutive overexpression of the *Drosophila* *period* protein inhibits *period* mRNA cycling. EMBO J 13:3590–3598.

3.	So WV, Rosbash M (1997) Post-transcriptional regulation contributes to *Drosophila* clock gene mRNA cycling. EMBO J 16:7146–7155.

4.	Bae K, Lee C, Sidote D, Chuang KY, Edery I (1998) Circadian regulation of a *Drosophila* homolog of the mammalian *Clock* gene: PER and TIM function as positive regulators. Mol Cell Biol 18:6142–6151.

5.	Bae K, Lee C, Hardin PE, Edery I (2000) dCLOCK is present in limiting amounts and likely mediates daily interactions between the dCLOCK-CYC transcription factor and the PER-TIM complex. J Neurosci 20:1746–1753.

6.	Kim EY, Bae K, Ng FS, Glossop NR, Hardin PE, *et al.* (2002) *Drosophila* CLOCK protein is under posttranscriptional control and influences light-induced activity. Neuron 34:69–81.

7.	Marrus SB, Zeng H, Rosbash M (1996) Effect of constant light and circadian entrainment of *per* flies: evidence for light-mediated delay of the negative feedback loop in *Drosophila*. EMBO J 15:6877–6886.

8.	Lee C, Bae K, Edery I (1998) The *Drosophila* CLOCK protein undergoes daily rhythms in abundance, phosphorylation, and interactions with the PER-TIM complex. Neuron 21:857–867.

9.	Sehgal A, Rothenfluh-Hilfiker A, Hunter-Ensor M, Chen Y, Myers MP, *et al.* (1995) Rhythmic expression of *timeless*: a basis for promoting circadian cycles in *period* gene autoregulation. Science 270:808–810.

10.	Blau J, Young MW (1999) Cycling *vrille* expression is required for a functional *Drosophila* clock. Cell 99:661–671.

11.	Cyran SA, Buchsbaum AM, Reddy KL, Lin MC, Glossop NR, *et al.* (2003) *vrille*, *Pdp1*, and *dClock* form a second feedback loop in the *Drosophila* circadian clock. Cell 112:329–341.

12.	Glossop NR, Houl JH, Zheng H, Ng FS, Dudek SM, *et al.* (2003) VRILLE feeds back to control circadian transcription of *Clock* in the *Drosophila* circadian oscillator. Neuron 37:249–261.
